# The effect of two different types of forces on possible root resorption in relation to dentin phosphoprotein levels: a single-blind, split-mouth, randomized controlled trial

**DOI:** 10.1186/s40510-021-00388-y

**Published:** 2021-12-20

**Authors:** Sherifa Ghaleb, Nazla Tamish, Walid ElKenany, Myriam Guindi

**Affiliations:** 1 Department of Orthodontics, Faculty of Dentistry, Alexandria University, Champolion street, Azarita, Egypt; 2 Department of Clinical Pathology, Faculty of Medicine, Alexandria University. Champolion Street, Azarita, Egypt

**Keywords:** Root resorption, Dentine phosphoprotein, Gingival crevicular fluid, Continuous force, Intermittent force

## Abstract

**Background:**

The purpose of this 2-arm-parallel split-mouth trial was to evaluate and compare the extent of possible root resorption using dentin phosphoprotein levels in gingival crevicular fluid between controlled continuous and intermittent orthodontic force groups.

**Materials and methods:**

A sample of 16 maxillary first premolars from 8 patients requiring bilateral extractions of the upper first premolars as part of their orthodontic treatment were recruited. A buccally directed continuous force of 150 g, reactivated after 28 days, was applied to the upper first premolar on one side for 8 weeks. On the contralateral first premolar, a buccally directed intermittent force (21 days on, 7 days off) of the same magnitude was applied for the same period. Gingival crevicular fluid samples were collected at the beginning of the study, 1st, 3rd, 4th and 5th week, and at the end of the study to quantify and compare dentin phosphoprotein levels in both groups.

**Results:**

Dentin phosphoprotein levels showed a higher concentration in the continuous force group than the intermittent force group in week 4 and 8 of sample collection; where the differences were statistically significant (95% CI 0.007–0.14; *P* < .04) and (95% CI 0.02–0.17; *P* < .04) respectively. No harm was observed.

**Conclusions:**

Dentin phosphoprotein was found to be a useful early biomarker to detect and monitor root resorption, showing that the application of an intermittent orthodontic force caused less root resorption than a continuous force.

***Trial registration*:**

NCT04825665 ClinicalTrials.gov. Registered 1 April 2021—Retrospectively registered, https://clinicaltrials.gov/ct2/show/NCT04825665.

## Background

One of the unfavorable sequelae of orthodontic treatment is root resorption [1]. Root resorption can be defined as a pathological or physiological process that leads to loss of cementum and dentine [2]. Commonly, in orthodontics, it is referred to as an induced sterile inflammatory response which is a form of pathological external root resorption [1, 3]. When orthodontic forces are transferred to the teeth, hyalinized areas in the periodontal spaces are formed. Thereafter, cells and blood vessels from the adjacent healthy periodontium lead to removal of the hyalinized tissues, this eventually results in loss of both cementoid and mature collagen, adjacent to the cementum. Subsequently, changes of the normal barriers to root resorption occur [2, 4]. However, root resorption resulting from orthodontic treatment is usually mild and clinically insignificant; although in some patients it can occur in large amounts [5].

Factors that have been linked to root resorption are genetic characteristics, biological factors, age of the patient and orthodontic treatment techniques. Orthodontic treatment techniques include the magnitude, duration and type of orthodontic force application [6–11]. In relevance to the manner of orthodontic force application, current data suggest that pausing orthodontic forces during treatment may reduce the amount of root resorption. This is likely due to cementum repair during the inactive periods [8, 9]. Additionally, this is specifically important in individuals who are biologically and genetically prone to root resorption.

Early detection of teeth at risk of severe resorption is crucial [12]. Currently, using radiographs is common; however, they are technique sensitive, cannot indicate if the process of root resorption is still active and additional radiation exposure to the patient is a risk. Therefore, a safer, more reliable alternative method to clinically diagnose early stages of root resorption is much needed and may include detecting specific biomarkers in gingival crevicular fluid [13].

Gingival Crevicular Fluid (GCF) is the inflammatory transudate that flows out via the gingival crevice, known to contain an array of biochemical and cellular factors that reflect the state of the underlying periodontium [14]. Among the dentin breakdown products found in the GCF, that reflect loss of root structure is dentin phosphoprotein. Dentin phosphoprotein is a predominant non-collagenous protein that is cleaved from dentin sialophosphoprotein. Moreover, it accounts for approximately 50% of all the dentin non-collagenous proteins [15, 16].

### Specific objectives or hypotheses

In this study the aim was to compare the extent of root resorption between continuous and intermittent orthodontic force groups, using the levels of dentin phosphoprotein in gingival crevicular fluid.

## Methods

### Trial design

This was a single-blind randomized controlled trial. It had a 2-arm split-mouth design in which the maxillary right first premolar of each patient was randomly allocated to either the continuous or intermittent group using randomization software (www.randomization.com) with a 1:1 allocation ratio, while the maxillary left first premolar was assigned to the other group. A split-mouth design was employed to control any potential patient-related confounders such as root lengths, shapes, and age of patient. There were no alterations after commencement of the trial.

### Participants, eligibility criteria and settings

Ethics approval was obtained from the scientific research ethics committee of Faculty of Dentistry, Alexandria University and informed oral and written consents were obtained from the patients’ guardians. 

Sixteen maxillary first premolars from eight participants (3 boys and 5 girls) aged between 13 and 18 years (mean age 15.8 years) who were recruited from the Outpatient Clinic at the Department of Orthodontics, Faculty of Dentistry, Alexandria University. These participants required bilateral maxillary first premolar extractions as part of their orthodontic treatment. Patient recruitment commenced in March 2020 and ended in December 2020. No previous reported or observed trauma, dental treatment or orthodontic treatment were considered to be the selection criteria for the teeth to be extracted. Subjects were excluded if they had past or present signs or symptoms of periodontal disease or bruxism, medical history that would affect the dentition and if apexification was not completed.

### Interventions

**Fig. 1:**
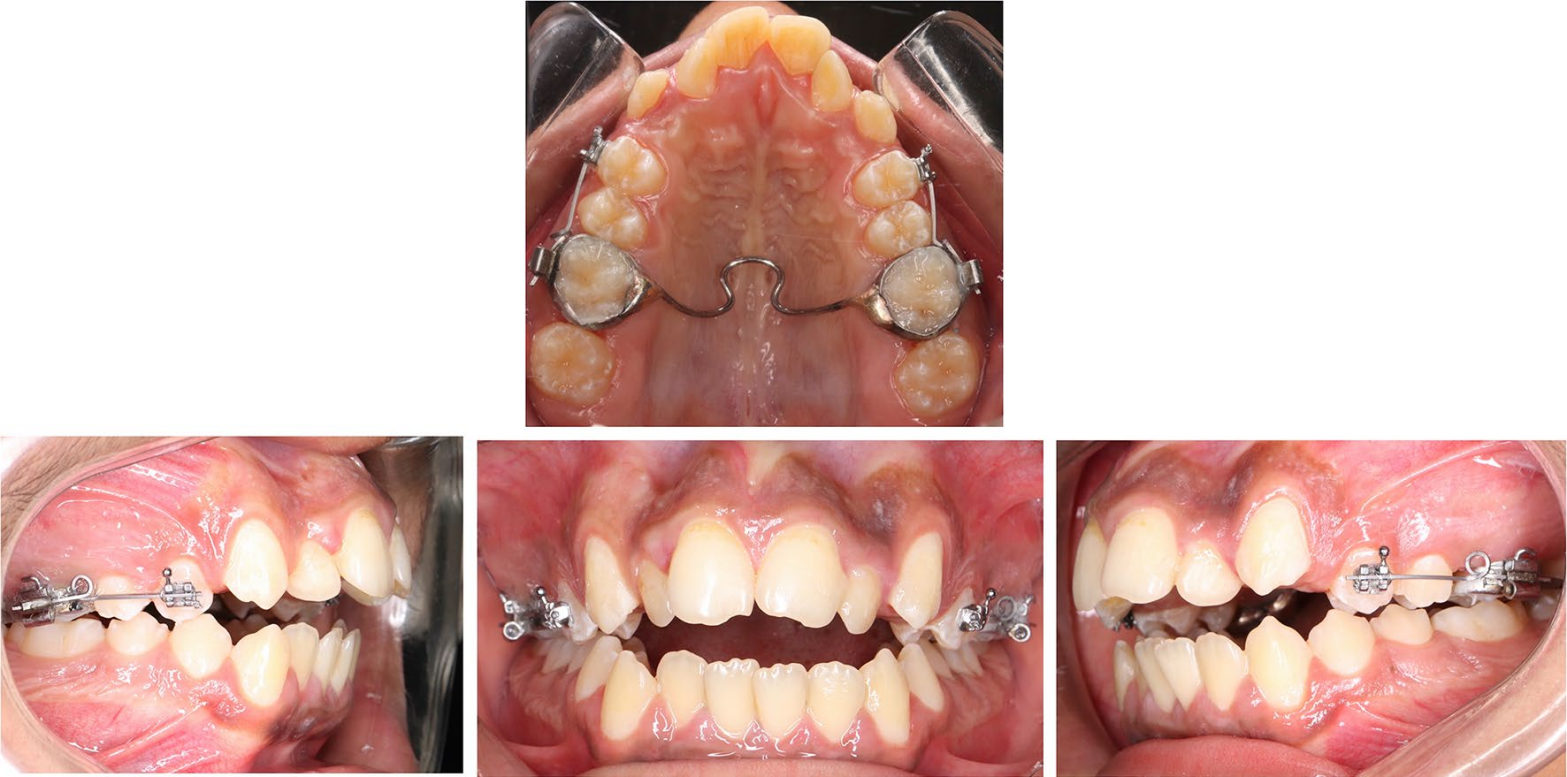
Experimental appliance

**Fig. 2:**
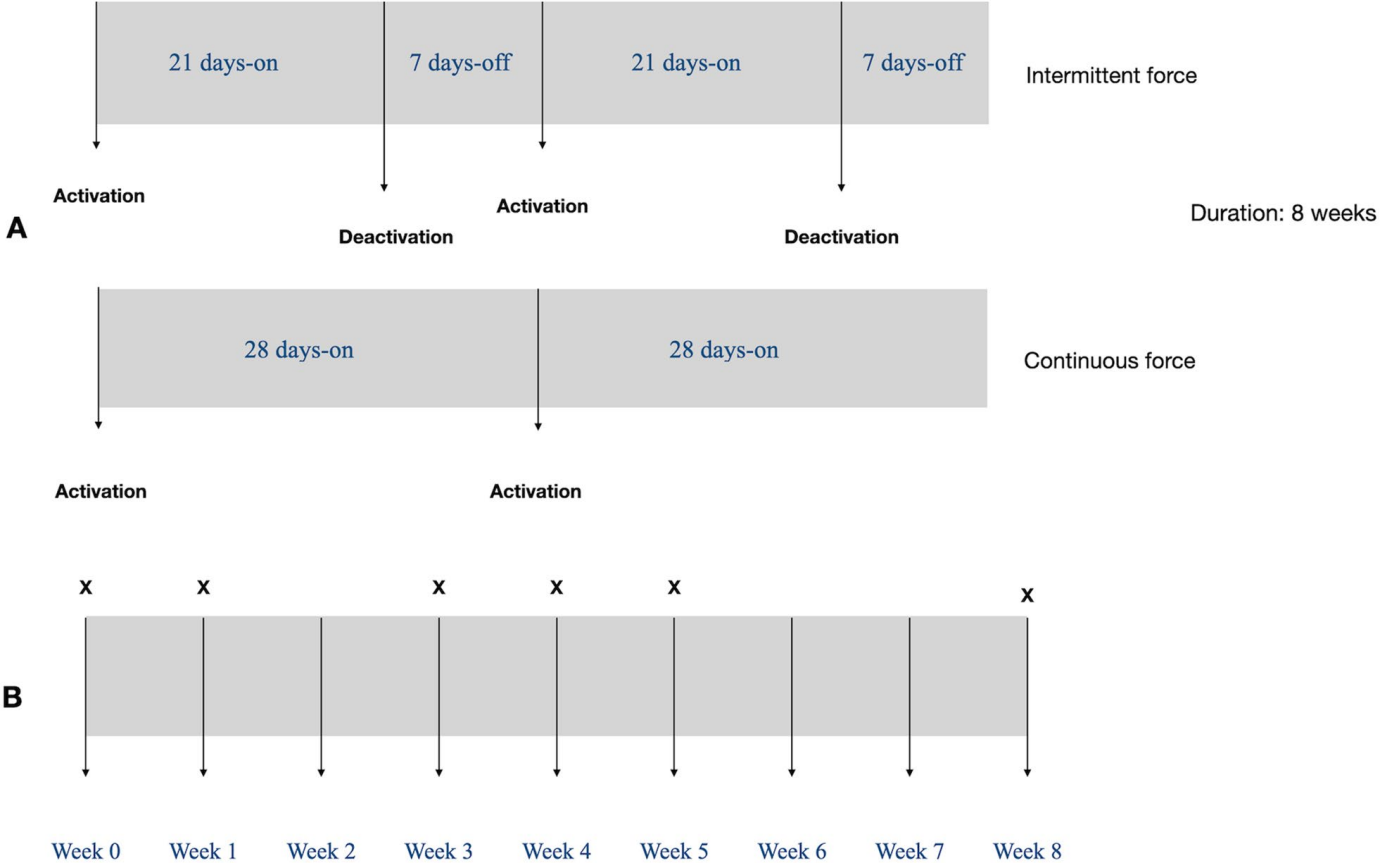
**a** Force application periods, **b** time points for collection of gingival crevicular fluid

In each subject, a buccally directed continuous tipping force of 150 g was applied to the maxillary first premolar (MFP) on one side for 8 weeks. On the contralateral side, a buccally directed intermittent force (21 days on, 7 days off) of the same magnitude was also applied for 8 weeks. The type of force application was assigned randomly to eliminate any allocation bias. A transpalatal arch was banded on the upper first molars to enhance the molar anchorage unit and ensure that the force applied to each experimental premolar was consistent. A 0.022-in slot premolar bracket (Mini 2000, Ormco, California, United States) was bonded on the buccal surfaces of each MFP. The forces were induced using 0.017″ × 0.025″ Titanium molybdenum alloy (TMA) cantilever spring (Beta III Titanium, 3 M Unitek, Monrovia, Calif) and the force magnitude was measured with a strain gauge (Morelli Ortodontia, Sorocaba, Brazil) (Fig. 1). On the continuous force side, the TMA springs were kept in place, checked and reactivated to the original force level on the 28th day of the experiment. While on the intermittent force side, a passive 0.017″ × 0.025″ TMA wire (Beta III Titanium, 3 M Unitek, Monrovia, Calif) was placed during the rest periods to maintain the position of the upper first premolar. At the end of the 7 day rest period, the springs were placed again and the force level was calibrated to the original amount (Fig. 2).

Samples were collected from both experimental sites at the beginning of the study, 1st week, 3rd week, 4th week, 5th week and at the end of the study period which is the 8th week (Fig. 2). Before the sample collection, each experimental tooth was isolated using a self-retaining retractor, suction, and cotton rolls, and gently dried with air for 5 s. Standardized filter paper strips (Whatman International Ltd, Maidstone, UK), cut into standardized sizes 2 × 10 mm were used for the collection of the gingival crevicular fluid samples. Each strip was inserted into the gingival crevice until mild resistance was felt and left insitu for 60 s. After removal, new strips were placed at 1 min interval to obtain four strips at each site. Each set of 4 filter paper strips collected from the same experimental site were placed in an eppendorf tube containing 100 ul of phosphate-buffered saline. It was then centrifuged at 3000×*g* for 10 min and stored at − 20 °C for later analysis. Samples contaminated with saliva or blood were discarded and new samples were collected.

The samples were then assayed for dentin phosphoprotein using a dentin phosphoprotein (DPP) assay kit (Cloud clone, USA). Samples were analyzed using the enzyme linked immunosorbent assay (ELISA) method. The concentration of DPP in the samples was determined by comparing the optical density (O.D) of the samples to the standard curve. According to standards’ concentrations and the corresponding OD values, the linear regression equation of the standard curve was calculated. Final results were reported as dentin phosphoprotein level ng/ml per 60-s sample [17].

At the end of the experimental period, the experimental teeth were extracted with instructions to avoid forceps contact on the cervical cementum. They were immediately stored in marked tubes containing deionized water. The periodontal ligament was removed by using an ultrasonic bath for 10 min, followed by disinfection in 70% alcohol for 30 min, and then stored again in the deionized water. Teeth were bench dried before imaging [9].

**Fig. 3:**
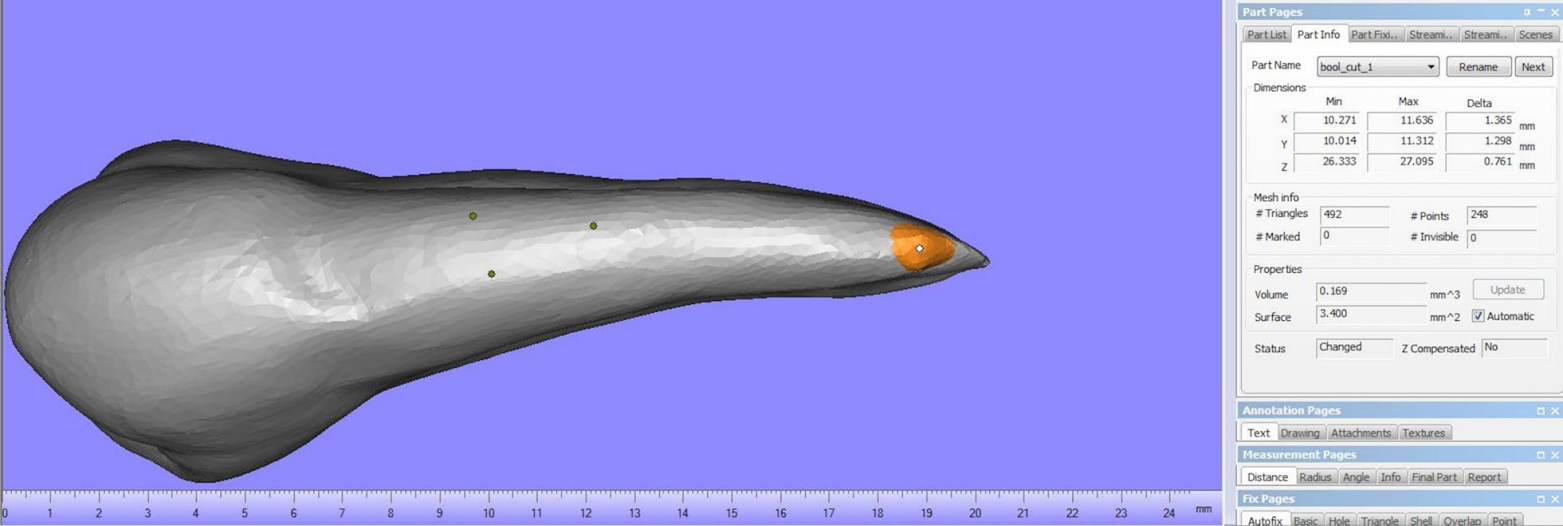
Resorption crater

The extracted teeth were scanned by a high resolution cone beam computed tomography system (Vatech Green CT, Korea) under a voxel size of 80 microns, a voltage of 95 kilovolts and current of 7 milliampere. After acquisition, the software ImageJ (ImageJ, National Institute of Health, USA) was used to measure the area of the root resorption craters. As for the volume estimation, Ondeman3dApp (Cybermed, Korea) was used for 3D STL file exporting, Exocad (Darmstadt, Germany) was used in reconstructing the resorption crater with a brush tool [18] and then lastly Materialise Magics 18.03 (Materialise, Belgium) was used to calculate the estimated volume of each resorption crater. This was done by measuring the volume of the tooth with the reconstructed crater and the original non constructed tooth, then subtracting them from each other to get the estimated volume of each resorption crater. Each resorption crater’s position on the tooth was recorded (Fig. 3).

**Fig. 4:**
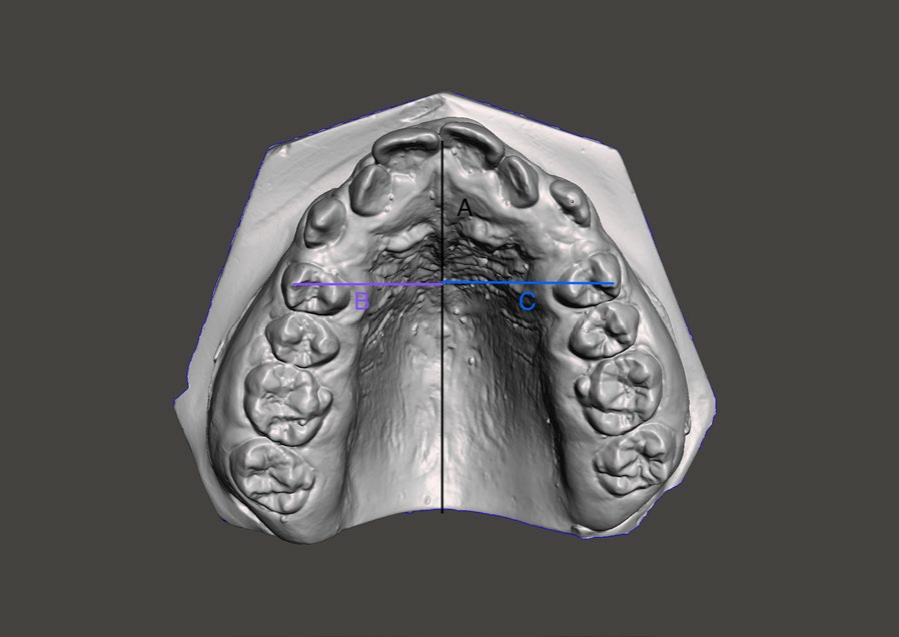
Measurement of buccal movement of premolars. **a** Line demonstrating median palatine raphe; distances of (**b**), the right premolar and **c** the left premolar to the (**a**) line

Maxillary dental plaster models were obtained before and after the experimental period and were scanned with an inEos X5 scanner (Dentsply, Sirona, USA). A reference line passing through the median palatine raphe was then constructed. The distances of the buccal cusps of the first premolars to the reference line were measured to calculate the buccal movement of each premolar and then comparison between both force groups was done (Fig. 4).

### Outcomes (primary and secondary) and any changes after trial commencement

The main outcome of this study was to evaluate and compare the extent of root resorption between the continuous and intermittent orthodontic force groups using the levels of dentin phosphoprotein in gingival crevicular fluid. The secondary outcome was the three-dimensional comparison of the root resorption craters’ volume and area in the extracted maxillary first premolar roots between both groups. The tertiary outcome was the comparison of amount of tooth movement during buccal tipping between both groups. There were no outcome changes after trial commencement.

### Sample size calculation

Sample size was estimated based on the following assumptions: alpha error = 5% and study power = 80%. According to Ballard et al. [9] the mean cube root resorption volume after orthodontic movement = 0.815, standard deviation (SD) calculated from the standard error = 0.099 when intermittent orthodontic force was used, and mean = 0.985, SD = 0.099 when continuous force was used. Dentin phosphoprotein in GCF is a biomarker for monitoring root resorption [13, 15]. Based on comparison of means, sample size was calculated to be 7 per group and this was increased to 8 to make up for cases lost to follow-up. Therefore total sample size = 16 [19].

### Randomization and blinding

The right first maxillary premolar from each patient was randomly allocated to either the continuous or intermittent group using randomization software (www.randomization.com) with a 1:1 allocation ratio. The left first maxillary premolar was then assigned to the alternate group. Allocation concealment was achieved with sequentially numbered, opaque, sealed envelopes containing the treatment allocation cards, which were prepared before the trial. The primary investigator was responsible for opening the next envelope in sequence and implementing the randomization process. Blinding of either patient or operator was not possible; however, blinding during outcome assessment was. This was because it was difficult to distinguish between the treatment groups during outcome assessment from the eppendorf tubes collected, the teeth scanned or the models analyzed.

### Statistical analysis

Normality was checked for all variables using descriptive statistics, normality tests and plots (boxplot and histogram). All variables showed non-normal distribution, so non-parametric analysis was adopted. Comparison of biomarker level, amount of tooth movements and crater volume between the two study groups was done using Wilcoxon signed rank test. Mean differences and 95% confidence intervals (CI) were calculated. Comparison of different timepoints for the biomarker level, and different regions of craters within each group were done using Friedman test, followed by multiple pairwise comparisons using Bonferroni adjusted significance level. Spearman correlation was performed to assess the relation between DPP levels at 8 weeks and total amount of root resorption in the two study groups. Significance was set at *P* value < 0.05 and data were analyzed using IBM SPSS for Windows (Version 23.0).

## Results

### Participant flow

**Fig. 5:**
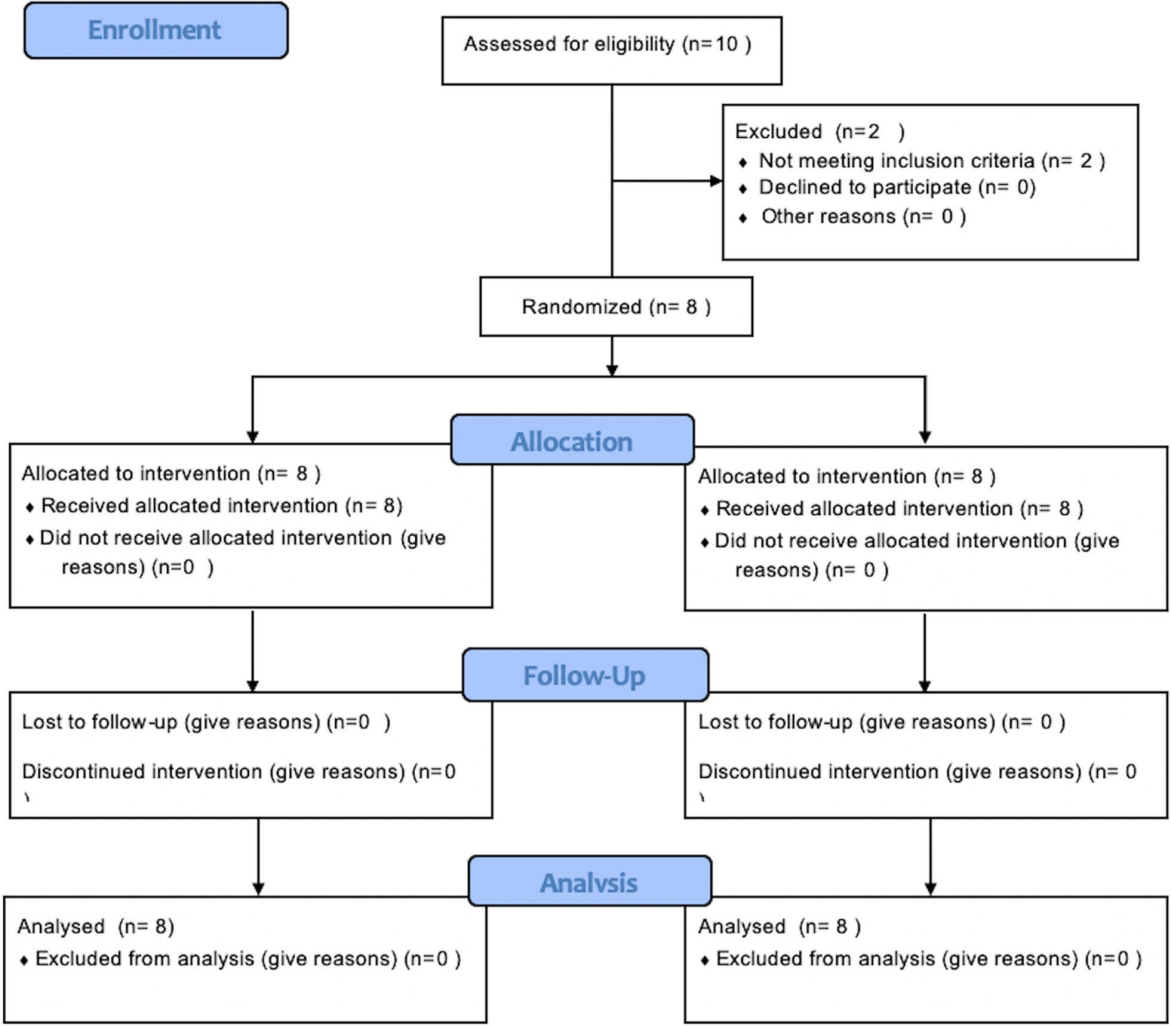
CONSORT flow chart showing patient flow during the trial

Patient flow through the study is illustrated in the CONSORT diagram (Fig. 5). Sixteen maxillary first premolars from eight orthodontic patients, which included 3 males (33%), 5 females (66%) with a mean age of (15.88 ± 2.03) years were randomized in a 1:1 ratio to either continuous or intermittent force group in the same patient. No patients were lost to follow-up. At baseline, at week 0, dentin phosphoprotein levels were similar on both sides (0.021 ± 0.009 and 0.020 ± 0.008 ng/ml per 60-s sample).

### Numbers analyzed for each outcome, estimation and precision, subgroup analyses

**Fig. 6:**
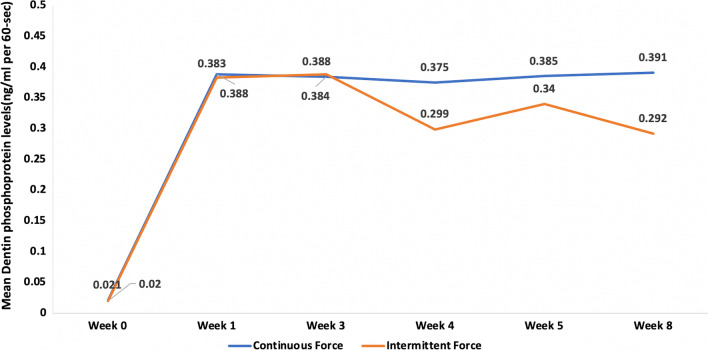
Dentin phosphoprotein biomarker levels at different time points in the two study groups

The sixteen maxillary first premolars randomly assigned to each group were all included in the analyses done. Dentin phosphoprotein levels showed a mean difference change from week 0 to week 8 of (0.370 ± 0.01) and (0.273 ± 0.09) in the continuous and intermittent groups respectively. However, it was higher in the continuous group with a statistical significant difference (95% CI 0.02–0.18; *P* < 0.05). DPP levels showed a statistically significant higher concentration in the continuous group than the intermittent group in weeks 4 and 8 (*P* < 0.05) (Table 1) (Fig. 6).

**Table 1:** Dentin phosphoprotein biomarker levels in the two study groups at the different time points

	Continuous force	Intermittent force	Mean difference (SD)	95% CI	WSR *P* value
*Week 0*					
Mean ± SD	0.021 ± 0.009	0.020 ± 0.008	0.001 (0.01)	− 0.01, 0.01	0.78
Median (IQR)	0.021 (0.012, 0.030)	0.021 (0.011, 0.028)			
*Week 1*					
Mean ± SD	0.388 ± 0.007	0.383 ± 0.005	0.005 (0.006)	− 0.0007, 0.01	0.13
Median (IQR)	0.386 (0.383, 0.387)	0.383 (0.382, 0.385)			
*Week 3*					
Mean ± SD	0.384 ± 0.003	0.388 ± 0.005	− 0.004 (0.006)	− 0.009, 0.0009	0.08
Median (IQR)	0.383 (0.383, 0.385)	0.389 (0.387, 0.392)			
*Week 4*					
Mean ± SD	0.375 ± 0.002	0.299 ± 0.08	0.07 (0.08)	0.007, 0.14	0.04*
Median (IQR)	0.375 (0.374, 0.375)	0.294 (0.267, 0.368)			
*Week 5*					
Mean ± SD	0.385 ± 0.004	0.340 ± 0.07	0.04 (0.07)	− 0.01, 0.10	0.21
Median (IQR)	0.385 (0.382, 0.388)	0.367 (0.263, 0.394)			
*Week 8*					
Mean ± SD	0.391 ± 0.002	0.293 ± 0.09	0.10 (0.09)	0.02, 0.17	0.04*
Median (IQR)	0.391 (0.390, 0.392)	0.292 (0.202, 0.392)			
*Mean difference (week 8–0)*					
Mean ± SD	0.370 ± 0.01	0.273 ± 0.09	0.10 (0.09)	0.02, 0.18	0.04*
Median (IQR)	0.372 (0.360, 0.380)	0.271 (0.180, 0.373)			
Friedman test	< 0.001*	< 0.001*			

*WSR* Wilcoxon signed rank test, *SD* standard deviation, *CI* confidence interval

*Statistically significant at *P* value < 0.05

Intragroup comparisons for both groups revealed that DPP levels at all time points compared to week 0 were higher, with a statistical significant difference in week 1, 3, 5 for both groups. While week 8 (0.391 ± 0.002) was statistically significantly higher than week 0 (0.021 ± 0.009) (*P* < 0.001) for the continuous group only. Also for the continuous group only, week 8 was statistically significantly higher than week 4 (*P* < 0.01) (Table 2).

**Table 2:** Post-hoc pairwise comparisons of biomarker levels in each group using Bonferroni adjusted significance levels

Time point	Compared to	Continuous force *P* value	Intermittent force *P* value
Week 0	Week 1	0.006*	0.008*
	Week 3	0.049*	< 0.001*
	Week 4	1.00	0.68
	Week 5	0.008*	0.01*
	Week 8	< 0.001*	0.14
Week 1	Week 3	1.00	1.00
	Week 4	0.20	1.00
	Week 5	1.00	1.00
	Week 8	1.00	1.00
Week 3	Week 4	0.92	0.17
	Week 5	1.00	1.00
	Week 8	0.58	0.79
Week 4	Week 5	0.24	1.00
	Week 8	0.001*	1.00
Week 5	Week 8	1.00	1.00

*Statistically significant difference using Bonferroni adjusted significance level

**Fig. 7:**
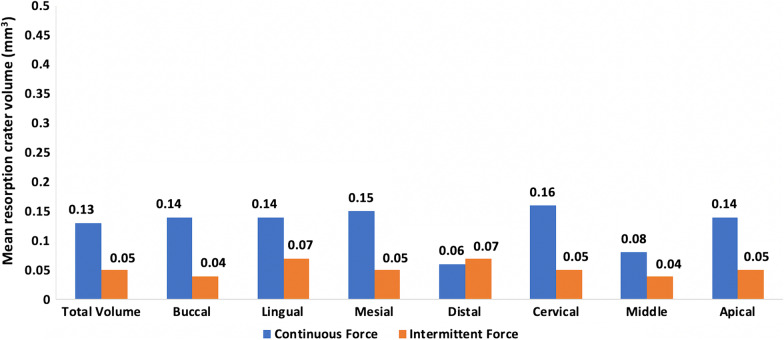
Mean root resorption crater volumes in the two study groups

The mean resorption crater area for the continuous group was higher than the intermittent force group with a statistical significant difference (*P* < 0.05). The mean resorption crater volume (MRCV) for the continuous force group (0.13 ± 0.06 mm^3^) was higher than the intermittent force group (0.05 ± 0.03mm^3^). This difference was statistically significant as well (95% CI, 0.06–0.10; *P* < 0.001) (Table 3) (Fig. 7).

**Table 3:** Root resorption craters area and volume in the two study groups

	Continuous force	Intermittent force	Mean difference (SD)	95% CI	WSR *P* value
	Mean ± SD				
Crater area	1.92 ± 0.82	1.48 ± 0.34	0.43 (1.01)	0.06, 0.08	0.03*
Total crater volume	0.13 ± 0.06	0.05 ± 0.03	0.08 (0.06)	0.06, 0.10	< 0.001*
Total buccal volume	0.14 ± 0.07	0.04 ± 0.03	0.10 (0.07)	0.05, 0.14	0.003*
Total lingual volume	0.14 ± 0.02	0.07 ± 0.04	0.07 (0.06)	− 0.02, 0.16	0.07
Total mesial volume	0.15 ± 0.05	0.05 ± 0.04	0.11 (0.06)	0.05, 0.18	0.03*
Total distal volume	0.06 ± 0.001	0.07 ± 0.02	− 0.02 (0.03)	− 0.26, 0.22	0.18
Total cervical volume	0.16 ± 0.06	0.05 ± 0.04	0.11 (0.07)	0.06, 0.15	0.002*
Total middle volume	0.08 ± 0.02	0.04 ± 0.02	0.04 (0.03)	− 0.03, 0.10	0.11
Total apical volume	0.14 ± 0.02	0.05 ± 0.02	0.09 (0.03)	0.06, 0.12	0.03*
*Friedman test*					
Buccal–lingual–mesial–distal	0.31	0.20			
Cervical–middle–apical	0.10	0.69			

*WSR* Wilcoxon signed rank test, *SD* standard deviation, *CI* confidence interval

*Statistically significant at *P* value < 0.05

Results for the different tooth surfaces showed that the MRCVs on the buccal and mesial surfaces especially were statistically significantly greater in the continuous force group (*P* < 0.01) and (*P* < 0.05) respectively. While the results for different root levels showed that at the cervical and apical thirds, the continuous force group displayed greater MRCVs with a statistically significant difference, (*P* < 0.01) and (*P* < 0.05) respectively (Table 3) (Fig. 7). The buccal cervical region was the only region which displayed statistically significantly higher MRCVs in the continuous force group compared to the intermittent force group (*P* < 0.01) (Table 4).

**Table 4:** Root resorption crater volume at different regions in the two study groups

	Continuous force	Intermittent force	Mean difference (SD)	95% CI	WSR *P* value
	Mean ± SD				
*Buccal*					
Cervical	0.15 ± 0.08	0.05 ± 0.03	0.10 (0.08)	0.03, 0.16	0.008*
Middle	0.06 ± 0.008	0.005 ± 0.0007	0.06 (0.007)	− 0.003, 0.12	0.18
Apical	0.06 ± 0.002	0.06 ± 0.001	0.0002 (0.0007)	− 0.004, 0.009	0.18
P value	0.16	0.22			
*Lingual*					
Cervical	0.16 ± 0.02	0.11 ± 0.001	0.05 (0.02)	− 0.16, 0.25	0.18
Middle	0.14 ± 0.00	0.004 ± 0.00	0.14 (0.00)	− 0.001, 0.001	0.18
Apical	0.14 ± 0.02	0.03 ± 0.002	0.12 (0.03)	− 0.12, 0.35	0.18
*P* value	0.37	0.37			
*Mesial*					
Cervical	0.18 ± 0.02	0.06 ± 0.06	0.14 (0.05)	− 0.06, 0.21	0.07
Middle	0.09 ± 0.0007	0.05 ± 0.04	0.05 (0.02)	− 0.12, 0.22	0.18
Apical	0.06 ± 0.001	0.06 ± 0.02	0.0004 (0.03)	0.25, 0.26	0.66
*P* value	0.14	0.17			
Distal					
Cervical	0.12 ± 0.01	0.11 ± 0.001	0.01 (0.02)	− 0.13, 0.15	0.18
Middle	0.06 ± 0.001	0.05 ± 0.001	0.001 (0.00)	− 0.01, 0.01	0.16
Apical	0.07 ± 0.001	0.07 ± 0.0007	0.001 (0.002)	− 0.02, 0.02	0.32
*P* value	0.14	0.14			

*WSR* Wilcoxon signed rank test, *SD* standard deviation, *CI* confidence interval

*Statistically significant at *P* value < 0.05

There was a moderate direct significant correlation between DPP levels at 8 weeks and total root resorption volume in the continuous force group (Rho = 0.44, *P* = 0.01), and a weak direct correlation between DPP levels and total root resorption in the intermittent force group (Rho = 0.36, *P* = 0.02) (Table 5).

**Table 5:** Correlation between DPP level at 8 weeks and total root resorption volume in the two study groups

	Correlation coefficient (Rho)	*P* value
Continuous force	0.44	0.01*
Intermittent force	0.36	0.02*

*Statistically significant at *P* value < 0.05

The amount of buccal tooth movement was statistically significantly greater in the continuous force group compared with the intermittent force group with a mean difference of 1.13 (*P* < 0.05) (Table 6).

**Table 6:** Amount of tooth movement during buccal tipping in the two study groups

	Continuous force	Intermittent force	Mean difference (SD)	95% CI	WSR *P* value
Mean ± SD	2.13 ± 1.55	1.00 ± 0.84	1.13 (1.19)	0.13, 2.12	0.03*
Median (IQR)	2.00 (0.50, 3.75)	0.75 (0.50, 1.75)			

*WSR* Wilcoxon signed rank test, *SD* standard deviation, *CI* confidence interval

*Statistically significant at *P* value < 0.05

### Harms

There were no reported harms or adverse effects.

## Discussion

Commonly, radiographs are used to diagnose root resorption. However, it was found that 60–70% of mineralized tooth structure would have already been lost. Additionally, radiographs are unable to indicate if the process of root resorption is still active and exposes patients to unnecessary radiation exposure [13]. Alternatively, biomarkers have been suggested, in limited studies, to be a reliable method to clinically diagnose early stages of root resorption. Thus this study evaluated and compared the possible effects of continuous and intermittent orthodontic forces on root resorption by assessing dentin phosphoprotein levels.

Results of a systematic review by Tarallo et al. [20] compared different biomarkers in the GCF to detect root resorption in patients undergoing orthodontic treatment. They concluded that DPP was the most specific biomarker for early diagnosis of root resorption and suggested it to be used in future clinical trials. Also, it was found that cementum proteins are not highly indicative of the actual root structure loss because some areas of the cementum can be resorbed and repaired during orthodontic tooth movement. Therefore, previous studies suggested that the most indicative markers for the diagnosis of root resorption were dentin specific proteins only. Proteins like dentin sialoprotein have been suggested to be less indicative, since they were found to be present in both the root cement matrix and the dentin [20, 21]. Consequently, dentin phosphoprotein was selected as the biomarker for this study.

Multiple findings in this study showed that DPP may be an excellent biomarker for detecting root resorption. Both groups, at week 1 and 3 when compared to week 0 had statistically significantly higher DPP levels. This could be explained by the start of root resorption once the 150 g force was applied. Moreover until week 3, dentin phosphoprotein levels increased in both force groups with no statistical significant difference between them, since both had the force adjusted at the same level (150 g) at the start. Furthermore, in weeks 4 and 8 and the mean difference calculated between week 8 and week 0, a statistically significant difference in the DPP level between the continuous and intermittent force group was found. This suggested that DPP could detect the difference in root resorption between the two different groups. This was in agreement with the study done by Mah et al. [15] which showed that there is a statistically significant difference between a control group, the roots of the second primary molars undergoing resorption and an orthodontic group. Balducci et al. [13] achieved similar results, where concentrations of DPP were highest in the group with severe root resorption followed by the mild resorption group and the control group.

By week 4, after the 7 day rest period for the intermittent group, the continuous force group exhibited a significantly higher level of DPP. This corroborated the fact that cementum has a repair potential when a pause of force occurs resulting in less progressive root resorption. Again, this was in agreement with several studies [8, 9, 22]. This also occurred at week 8, for the second cycle; suggesting that the intermittent force manner may be beneficial for certain patients who are at high risk of root resorption. At week 5, DPP in the intermittent group started to increase again since the force level was reactivated after 28 days for both groups. Presence of DPP at Week 0 when teeth were not subjected to any orthodontic forces yet, was explained previously by Balducci et al. [13] and Mah et al. [15]. They found that the most reasonable explanation to this finding could be attributed to the sensitivity of the ELISA method.

This intermittent regimen of a 7 day rest period was chosen in accordance to a previous study by Ozkalayci et al. [8] since a 3-day inactive period applied by two previous studies meant that the patients had to attend their orthodontic adjustment appointments more frequently, which could be inconvenient to the patients and clinicians [9, 23]. Moreover, from a biological standpoint, the longer rest period would allow more time for the recruitment of cementoblasts and initiation of the reparative process. Moreover, since generally clinicians see patients at 4- to 6-week intervals for their orthodontic adjustment, we opted for a 8 week (56 days) total study period in order to mimic a 4 week interval patient visit, repeating it over 2 cycles. This also accommodates for patients who might require more than 10 to 35 days for the resorption craters to appear as Ballard et al. [9] explained in their study with a 8 week study period as well.

In this study high resolution cone beam computed tomography was used which followed the suggestions of several previous studies that compared different voxel sizes of cone beam computed tomography (CBCT) in the diagnosis of external root resorption. High resolution CBCT with a smaller voxel size has been suggested to be used when one intends to investigate the early stages of external root resorption [24, 25]. Mean root crater area and MRCV in the intermittent force group when compared to the continuous force group were significantly less, which is in agreement with the results of other studies [8, 23]. This finding also suggests that the 1-week inactive period permits the reversible defense mechanism to initiate the reparative process on the root surface [26].

The MRCVs on the buccal surfaces and the mesial surfaces, specifically, were significantly greater in the continuous force group. This conformed with the results and explanation of another study which stated that the force direction produced by the spring led to buccal tipping movement which may have induced a pressure zone at the buccal side [23]. Over and above, the premolar morphology and the rotational nature of the cantilever spring, may have been the cause of the pressure zone detected on the mesial surface [8]. The buccal tipping movement may also explain the significantly higher MRCV at the buccal cervical region in the continuous force group. While the intermittent force group could have experienced all the previous, however it had the time to repair, explaining the significant difference.

Ozkalayci et al. [8] found that MRCV in the intermittent force group was greater at the apical level, while our study found the opposite. This may be explained by the passive wire placed during the 7-day inactive periods in our study in order to retain the buccally tipped maxillary first premolar in place, which was suggested but not placed by the other study. Hence, when applying intermittent force during orthodontic treatment, it is important to have a passive archwire in place during the inactive phases to avoid relapse and pressure zones at the apical area. Continuous force application resulted in greater tooth movement, as shown by the buccal cusp movement, which correlated with previous studies [8, 23].

### Limitations

The Systematic review by Tarallo et al. [20] suggested to design new clinical trials focusing on the ability of GCF to diagnose root resorption early and to prefer the DPP as a biomarker. Since the previous studies using DPP to detect root resorption were only two [13, 15]. This study is also only the beginning. Further investigations with a larger sample size and using more sensitive DPP kits, if produced in the future, will be beneficial to overcome the shortcomings of the present investigation and highlight the clinical relevance as well. Lastly, a shortcoming of this study may be due to salivary contamination of the GCF collected; however, special care was taken to discard any samples contaminated with saliva or blood and new samples were taken immediately.

### Generalizability

This study showed that dentin phosphoprotein in gingival crevicular fluid can help patients who are genetically and/or biologically prone to root resorption, since it can diagnose early root resorption and can monitor it throughout the orthodontic treatment. Also, for these patients in specific, the intermittent force regimen was found to be a convenient and effective way to move teeth with less damaging effects on the root surface.

## Conclusions

Based on the findings of this randomized clinical trial, it was concluded that:

1. Dentin phosphoprotein was found to be a useful biomarker for detecting and monitoring root resorption. Patients who are prone and predisposed to orthodontically induced inflammatory root resorption may benefit from the biomarker’s early, radiation free diagnostic ability.
2. Intermittent orthodontic force caused less root resorption than the continuous orthodontic force, in relevance to dentin phosphoprotein levels and mean volume estimation of root resorption craters.
3. The efficiency of tooth movement was compromised with the intermittent force group compared to the continuous force group.

## Data Availability

The datasets used and/or analyzed during the current study are available from the corresponding author on reasonable request.

## Abbreviations

GCF: Gingival Crevicular Fluid
MFP: Maxillary first premolar
TMA: Titanium molybdenum alloy
DPP: Dentin phosphoprotein
ELISA: Enzyme linked immunosorbent assay
O.D: Optical Density
SD: Standard deviation
CI: Confidence intervals
MRCV: Mean resorption crater volume
CBCT: Cone beam computed tomography
